# Patterns of response with talimogene laherparepvec in combination with ipilimumab or ipilimumab alone in metastatic unresectable melanoma

**DOI:** 10.1038/s41416-019-0530-6

**Published:** 2019-07-29

**Authors:** Jason Chesney, Igor Puzanov, Frances Collichio, Mohammed M. Milhem, Axel Hauschild, Lisa Chen, Anjali Sharma, Claus Garbe, Parminder Singh, Janice M. Mehnert

**Affiliations:** 1 James Graham Brown Cancer Center, University of Louisville, Louisville, KY USA; 2 Roswell Park Comprehensive Cancer Center, Buffalo, NY USA; 3 The University of North Carolina at Chapel Hill, Chapel Hill, NC USA; 4 University of Iowa Hospitals and Clinics, Iowa City, IA USA; 5 University of Kiel, Kiel, Germany; 6 Amgen Inc., Thousand Oaks, CA USA; 7 University Hospital Tübingen, Tübingen, Germany; 8 Mayo Clinic, Phoenix, AZ USA; 9 Rutgers Cancer Institute of New Jersey, New Brunswick, NJ USA

**Keywords:** Melanoma, Targeted therapies

## Abstract

Talimogene laherparepvec (T-VEC) has demonstrated efficacy for unresectable melanoma. We explored response patterns from a phase 2 study evaluating patients with unresectable stage IIIB–IVM1c malignant melanoma who received T-VEC plus ipilimumab or ipilimumab alone. Patients with objective response per modified irRC were evaluated for pseudo-progression (single ≥25% increase in tumour burden before response). Patients without pseudo-progression were classified by whether they responded within or after 6 months of treatment start; those with pseudo-progression were classified by whether pseudo-progression was due to increase in existing lesions or development of new lesions. Overall, 39% (*n* = 38/98) in the combination arm and 18% (*n* = 18/100) in the ipilimumab arm had an objective response. Eight responders (combination, *n* = 7 [18.4%]; ipilimumab, *n* = 1 [5.6%]) had pseudo-progression; most occurred by week 12 and were caused by an increase in existing lesions. These data reinforce use of T-VEC through initial progression when combined with checkpoint inhibitors.

**Trial Registration** NCT01740297 (ClinicalTrials.gov; date of registration, December 4, 2012); 2012-000307-32 (ClinicalTrialsRegister.eu; date of registration, May 13, 2014).

## Background

Evidence of delayed response or disease progression before response (i.e. pseudo-progression) has been seen with immunotherapies.^1–3^ Increases in baseline lesions may be attributed to T-cell infiltration rather than tumour cell proliferation,^1^ resulting in increased tumour size that is not truly tumour growth. Because of this observation, the immune-related response criteria (irRC)^1^ are frequently used to measure response in lieu of Response Evaluation Criteria in Solid Tumors (RECIST) version 1.1.^4^ Talimogene laherparepvec (T-VEC) monotherapy has also been shown to induce perceived tumour progression before response using RECIST version 1.1 criteria.^5^

Improvements in overall response rates and in lesion-level response rates were observed with T-VEC administered in combination with other therapies.^6,7^ In a randomised trial, T-VEC plus ipilimumab resulted in a significantly higher objective response rate (ORR) versus ipilimumab alone (odds ratio, 2.9; 95% Cl, 1.5–5.5; *P* = 0.002) in 198 patients with metastatic unresectable melanoma.^7^ Similarly, the phase 1 MASTERKEY-265 trial of T-VEC plus pembrolizumab resulted in a confirmed ORR (rate of complete or partial response) of 61.9% (95% CI, 38.4–81.9%) and a complete response (CR) rate of 33.3% (95% CI, 14.6–57.0%) in 21 patients with advanced melanoma.^6^

To better define clinically meaningful response patterns in patients receiving T-VEC with an immune checkpoint inhibitor, this exploratory analysis evaluated patterns of response in patients with melanoma enrolled in the phase 2 study (ClinicalTrials.gov identifier, NCT01740297) of T-VEC plus ipilimumab versus ipilimumab alone.

## Methods

### Patients and study design

The primary analysis of this phase 2, randomised, open-label study has been previously reported.^7^ In the analysis reported here, patients with an objective response were evaluated for pseudo-progression. Patients were randomised (1:1) to receive T-VEC plus ipilimumab or ipilimumab (Fig. S1)^7^ and stratified by disease stage (stage IIIB/IIIC/IVM1a versus IVM1b/IVM1c) and previous therapy (treatment-naive; previous systemic anticancer immunotherapy; systemic anticancer treatment other than immunotherapy).^7^ Study procedures were approved by institutional review boards/ethics committees; patients provided informed consent. Additional details are provided in Supplement.

### Assessments

Tumour response was assessed at baseline and every 12 weeks after treatment initiation using irRC until documentation of confirmed disease progression, determined as a repeat, consecutive disease progression ≥4 weeks after initial disease progression. Additional details are provided in Supplement.

### Statistical analysis

Study weeks were calculated from date of randomisation. Pseudo-progression was defined as disease progression before patient response; however, a confirmation for disease progression per the irRC was not required. Patients with pseudo-progression were further classified according to whether the pseudo-progression was caused by an increase in existing lesions or the development of new measurable lesions.

Patients without pseudo-progression were classified according to whether they responded within 6 months (≤183 days) or after 6 months (>183 days) following treatment start. Timing of response onset was calculated as the date of response onset minus the date of first dose plus one day.

## Results

### Patients

**Fig. 1:**
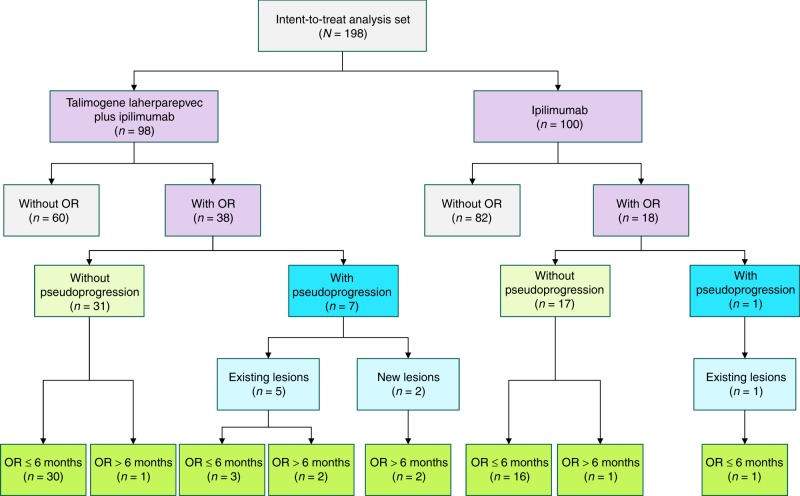
Patient incidence of pseudo-progression. OR objective response

Of 198 patients enrolled (T-VEC plus ipilimumab, *n* = 98; ipilimumab alone, *n* = 100), 56 (28%) had a confirmed objective response (T-VEC plus ipilimumab, *n* = 38 [39%]; ipilimumab, *n* = 18 [18%]; Fig. 1) and were included in the patterns of response analysis. Patient demographics and baseline clinical characteristics for patients with an objective response are shown in Table S1

.

### Patterns of response

Seven responders (18%) in the T-VEC plus ipilimumab arm and one responder (6%) in the ipilimumab alone arm had pseudo-progression (Table S2). Most cases of pseudo-progression were observed at the first tumour assessment at week 12 (*n* = 7/8; 88%). Of the eight patients with pseudo-progression, six experienced progression due to an increase in the size of existing lesions (T-VEC plus ipilimumab, *n* = 5; ipilimumab, *n* = 1; Fig. S2A, Fig. S2B), and two experienced progression due to the development of new measurable lesions leading to a total burden increase exceeding the 25% threshold for disease progression (T-VEC plus ipilimumab, *n* = 2; Fig. S2C). For all patients with pseudo-progression, median (range) duration of response (DOR) was 36.5 (12.0–123.7) weeks; median (range) DOR was 36.1 (12.0–101.0) weeks for T-VEC plus ipilimumab and 123.7 weeks for the patient in the ipilimumab arm. The patient in the ipilimumab arm received treatment for ~2 months and then underwent on-protocol surgery 2 months later. No subsequent anticancer therapy was received after surgery.

Forty-eight patients responded without pseudo-progression; 46 (96%) had an objective response within 6 months (T-VEC plus ipilimumab, *n* = 30; ipilimumab, *n* = 16; Fig. S3A, S3B) and two (4%) had an objective response after 6 months (T-VEC plus ipilimumab, *n* = 1; ipilimumab, *n* = 1; Fig. S3C, S3D). For these 46 patients, median (range) DOR was 47.9 (4.3–136.1) weeks; median (range) DOR was 49.0 (4.3–136.1) weeks for T-VEC plus ipilimumab and 46.7 (12.3–129.0) for ipilimumab alone. Patient response images are depicted in the Supplement (Fig. S4).

## Discussion

From our analysis, we identified 18% of patients receiving T-VEC plus ipilimumab and 6% of patients receiving ipilimumab alone who had pseudo-progression and eventually achieved a response. The incidence of pseudo-progression was higher in the combination arm and was associated with a higher ORR versus the control arm, 39% versus 18%, respectively. Because 18% of patients in the combination arm experienced pseudo-progression, we believe that T-VEC plus immune checkpoint inhibitors should be administered even in the setting of signs of progression to provide patients with the greatest opportunity to respond.

The incidence of pseudo-progression with T-VEC plus ipilimumab (18%) in our study was lower than observed with T-VEC monotherapy in OPTiM (48%),^5^ but higher than rates reported with other checkpoint inhibitor monotherapies (ipilimumab, 10%; pembrolizumab, 7%).^1,3^ Pseudo-progression rates in this study were higher than seen in an ipilimumab-nivolumab combination study^8^ and comparable to those observed in previous immunotherapy trials.^1–3^ DOR in this analysis was longer for patients without pseudo-progression versus those with pseudo-progression; median DOR was not reached for either group in the T-VEC monotherapy study.^5^ In most instances, pseudo-progression developed before week 12; however, cases of delayed pseudo-progression (e.g. after week 12) also occurred in this study (one of eight responders; 13%) and in previous trials.^1,3^ The timing of pseudo-progression does not appear to affect treatment response after progression.^1,3^ These data indicate that pseudo-progression is common with immune checkpoint inhibitor therapies, T-VEC, and T-VEC plus ipilimumab. Oncologists who administer these agents should be aware of this likely immune-mediated phenomenon and consider continuing treatment for ≥3 months assuming no clinical deterioration is observed.

In this analysis, ~15% of patients with an objective response overall had pseudo-progression, mostly due to an increase in existing lesion size. Because of this apparent tumour enlargement, RECIST version 1.1 (a ≥30% decrease in tumour burden measured in one dimension, longest diameter versus baseline^4^ would have captured this pseudo-progression as true progression; and the eight patients with pseudo-progression would likely have stopped T-VEC prematurely and their ultimate objective response would not have occurred. Thus, the data reported in this trial encourage the continued use of the revised irRC for evaluating immunotherapies and support the additional monitoring and treatment of patients through initial tumour progression.

Overall, these findings support the continued use of T-VEC, particularly when administered in combination with ipilimumab, through initial tumour progression in patients with melanoma, and use of irRC when evaluating response to immunotherapies to make the right treatment decisions for patients receiving chronic or maintenance administration of T-VEC and/or immune checkpoint inhibitors.

## Supplementary information

Supplemental Material

## Data Availability

Qualified researchers may request data from Amgen clinical studies. Complete details are available at the following: http://www.amgen.com/datasharing.
