# Supplementary material for: Patterns of response with talimogene laherparepvec in combination with ipilimumab or ipilimumab alone in metastatic unresectable melanoma
Source: Br J Cancer. 2019 Jul 29;121(5):417–20. doi: 10.1038/s41416-019-0530-6 (PMC6738060; doi:10.1038/s41416-019-0530-6)

Supplemental Material

Supplemental Methods

Patients and Study Design

In the combination arm, T-VEC was injected intralesionally on day 1 of week 1 (10^6 plaque-forming units [PFU]/mL), then on day 1 of week 4 and every 2 weeks thereafter (10^8 PFU/mL); ipilimumab (3 mg/kg) was administered intravenously every 3 weeks beginning day 1 of week 6 for up to four infusions.[2] In the ipilimumab arm, ipilimumab (3 mg/kg) was administered intravenously every 3 weeks beginning day 1 of week 1 for up to four infusions.[2] T-VEC treatment continued until patients had a CR, all injectable tumors had disappeared, confirmed disease progression per modified irRC was obtained, or patient intolerance.[2] Ipilimumab treatment continued for four infusions until confirmed disease progression per irRC or unacceptable ipilimumab-related toxicity.[2]

Assessments

Tumor assessments were made via measurement of cutaneous, subcutaneous, or nodal tumors with calipers or via radiographic imaging of the chest, abdomen, and pelvis performed by computed tomography, positron emission tomography/computed tomography, or magnetic resonance imaging. In the exploratory analysis, the data-derived disease progression was defined as an increase from the nadir in tumor burden ≥25% in the existing lesion burden or after adding in the occurrence of new measurable lesions; the occurrence of new lesions was not necessarily labeled as disease progression as long as the tumor burden threshold was not crossed. Tumor burden in this analysis was measured as the sum of the products of the two largest of perpendicular diameters of index lesions plus new, measurable lesions (up to 5 new cutaneous lesions and 10 visceral lesions, maximum 5 new lesions per organ).

Supplemental Results

Patterns of Response

Examples of patient responses are shown in **Figure S4**. Patient A, a healthy 75-year-old man with a history of cutaneous melanoma of the right shoulder, was diagnosed with recurrent, *BRAF* wild-type, subdermal metastasis of the right deltoid. Three months into receiving talimogene laherparepvec plus ipilimumab therapy, the size of the deltoid tumor had significantly decreased, and by 18 months into the treatment the palpable tumor was completely gone and only skin discoloration remained. One year later, the patient remained in remission. Patient B was 67 years old with stage IIIC melanoma of the left thigh and calf. After 24 weeks of treatment with talimogene laherparepvec plus ipilimumab the patient achieved a partial response, and continued to have improvement after 130 weeks of therapy.

Table S1. Demographics and Baseline Clinical Characteristics of Patients with an Objective Response

Characteristic, n (%)	T-VEC Plus Ipilimumab (n=38)	Ipilimumab (n=18)	Overall (n=56)
Sex			
Female	16 (42)	7 (39)	23 (41)
Male	22 (58)	11 (61)	33 (59)
Age, median (range), years	65 (30−93)	57 (23−82)	62 (23–93)
Race			
White	38 (100)	17 (94)	55 (98)
American Indian or Alaska native	0	1 (6)	1 (2)
ECOG performance status			
0	29 (76)	14 (78)	43 (77)
1	9 (24)	4 (22)	13 (23)
Disease substage			
IIIB	3 (8)	1 (6)	4 (7)
IIIC	13 (34)	8 (44)	21 (38)
IVM1a	6 (16)	2 (11)	8 (14)
IVM1b	10 (26)	3 (17)	13 (23)
IVM1c	6 (16)	4 (22)	10 (18)
BRAF status			
Mutant	12 (32)	11 (61)	23 (41)
Wild-type	26 (68)	6 (33)	32 (57)
Missing/unknown	0	1 (6)	1 (2)
Baseline HSV-1 status*			
Negative	11 (29)	6 (33)	17 (30)
Positive	22 (58)	5 (28)	27 (48)

Characteristic, n (%)	T-VEC Plus Ipilimumab (n=38)	Ipilimumab (n=18)	Overall (n=56)
Unknown	5 (13)	7 (39)	12 (21)
Previous surgery	37 (97)	18 (100)	55 (98)
Previous anticancer therapy	7 (18)	7 (39)	14 (25)
Previous ipilimumab therapy	0	0	0
Baseline LDH			
≤1× ULN	35 (92)	13 (72)	48 (86)
>1–2× ULN	1 (3)	4 (22)	5 (9)
>2× ULN	1 (3)	1 (6)	2 (4)
Unknown	1 (3)	0	1 (2)
Visceral disease at baseline	14 (37)	6 (33)	20 (36)
SPD[†] of all index lesions, median (range), mm^2	761 (49−6636)	272 (50−5139)	574 (49–6636)

Data are shown as n (%) unless otherwise stated.
ECOG=Eastern Cooperative Oncology Group; HSV=herpes simplex virus; LDH=lactate dehydrogenase; ULN=upper limit of the normal range; T-VEC=talimogene laherparepvec.
*HSV-1 immunoglobulin G antibody reference ranges: negative, < 0.91; positive, > 1.09.
[†]SPD refers to the sum of the products of the two largest perpendicular diameters.

Table S2. Patient Incidence of Pseudoprogression

Characteristic, n (%)	T-VEC Plus Ipilimumab (n=38)	Ipilimumab (n=18)
Pseudoprogression in existing lesions	5 (13)	1 (6)
Pseudoprogression with new lesions	2 (5)	0
No pseudoprogression (responded within 6 months)	30 (79)	16 (89)
No pseudoprogression (responded after 6 months)	1 (3)	1 (6)

T-VEC=talimogene laherparepvec.

Table S3. Listing of Study Sites and Institutional Review Boards/ Ethics Committees

Site Name	IRB/IEC Name
**United States**	
Allina Health System dba Virginia Piper Cancer Institute	Quorum Review Inc.
Baptist MD Anderson Cancer Center	Baptist Medical Center Institutional Review Board
Beverly Hills Cancer Center	Western Institutional Review Board
Columbia University Medical Center	Columbia University Medical Center Institutional Review Board
Gabrail Cancer Center, LLC	Western Institutional Review Board
Hematology Oncology Associates	Western Institutional Review Board
Icahn School of Medicine at Mount Sinai	Program for the Protection of Human Subjects, Icahn School of Medicine at Mount Sinai
Indiana University Melvin and Bren Simon Cancer Center	Indiana University Institutional Review Board
Lakeland Regional Cancer Center	Lakeland Regional Medical Center IRB
Mayo Clinic	Mayo Clinic Institutional Review Board
Medical University of South Carolina	Medical University of South Carolina, Office of Research Integrity IRB
Morristown Medical Center	Atlantic Health System IRB
Mount Sinai Comprehensive Cancer Center	Mount Sinai Medical Center
Rush University Medical Center	Rush University Medical Center IRB
Rutgers Cancer Institute of New Jersey	Western Institutional Review Board
Saint Louis University Hospital	Saint Louis University IRB
San Francisco Oncology Associates Medical Group Inc	Western Institutional Review Board
Sutter Pacific Medical Foundation	Western Institutional Review Board
The Angeles Clinic and Research Institute, West Los Angeles Office	Western Institutional Review Board
The Medical College of Wisconsin	Medical College of Wisconsin Froedtert Hospital Institutional Review Board
University of Arizona Cancer Center	Western Institutional Review Board
University of California at Los Angeles Medical Center	University of California at Los Angeles Office of Human Research Protection Program
University of Cincinnati Medical Center	University of Cincinnati IRB
University of Colorado Cancer Center	Colorado Multiple Institutional Review Board

University of Iowa Hospital and Clinics	University of Iowa IRB
University of Louisville James Graham Brown Cancer Center	University of Louisville IRB Human Subjects Protection Program Office
University of North Carolina at Chapel Hill	University of North Carolina at Chapel Hill, Office of Human Research Ethics
University of Southern California, Norris Comprehensive Cancer Center	University of Southern California, Health Sciences Institutional Review Board
University of Texas MD Anderson Cancer Center	University of Texas MD Anderson Cancer Center Institutional Review Board
University of Utah Huntsman Cancer Institute	University of Utah IRB
Vanderbilt University Medical Center	Vanderbilt University IRB
Virginia Commonwealth University Massey Cancer Center	Western Institutional Review Board
**France**	
Centre Hospitalier Régional Universitaire de Lille - Hôpital Claude Huriez	Comité de Protection des Personnes Nord Ouest IV
Centre Hospitalier Universitaire de Bordeaux - Hôpital Saint André	Comité de Protection des Personnes Nord Ouest IV
Centre Hospitalier Universitaire de Grenoble - Hopital Nord Michallon	Comité de Protection des Personnes Nord Ouest IV
Centre Hospitalier Universitaire de Nantes, Hôpital Hôtel Dieu	Comité de Protection des Personnes Nord Ouest IV
Hopital Saint Louis	Comité de Protection des Personnes Nord Ouest IV
**Germany**	
Universitätsklinikum Schleswig-Holstein	Ethik-Kommission der Medizinischen Fakultät der Christian-Albrechts-Universität zu Kiel
Universitätsklinikum Tübingen	Ethikkommission der Medizinischen Fakultät am Universitätsklinikum
Universitätsmedizin Göttingen - Georg-August-Universität	Ethikkommission der Medizinischen Fakultät am Universitätsklinikum

IRB/IEC=Institutional Review Boards/Ethics Committees.

Figure S1. **Study schema for the phase 2, randomized, open-label study.** CNS=central nervous system; ECOG=Eastern Cooperative Oncology Group; irRC=immune-related response criteria; IV=intravenous; ORR=objective response rate; OS=overall survival; mets=metastases; PD-1=programmed death-1; PFS=progression-free survival; PFU=plaque-forming unit; Q2W=every 2 weeks; Q3W=every 3 weeks; Q12W=every 12 weeks; R=randomization. *irRC described in Wolchok JD, et al. *Clin Cancer Res*. 2009;15(23):7412-7420. Reprinted with permission from Chesney J, et al. *J Clin Oncol*. 018;36(17):1658-1667.

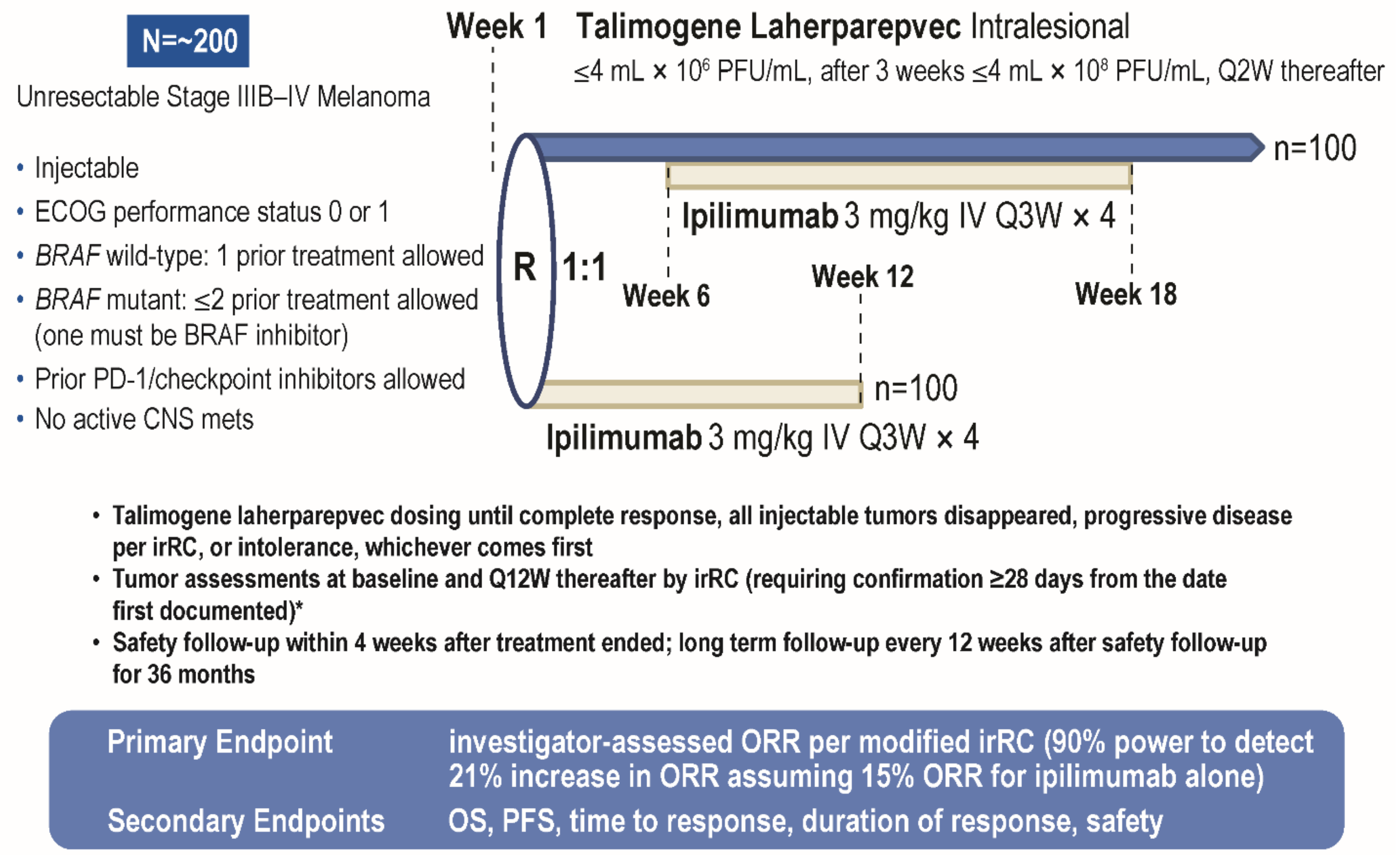

**Figure S2.	Patterns of response after treatment in patients with pseudoprogression. (A)** Patients treated with T-VEC plus ipilimumab who had pseudoprogression associated with an increase in existing lesions*; **(B)** patients treated with ipilimumab only who had pseudoprogression associated with an increase in existing lesions*; **(C)** patients treated with T-VEC plus ipilimumab who had pseudoprogression associated with the development of new lesions.

*Pseudoprogression due to increase in existing lesions meant tumor burden increase in existing lesions exceeded the threshold for progressive disease and does not imply that there were no new lesions.

Figure S2.

A. Pseudoprogression associated with an increase in existing lesions

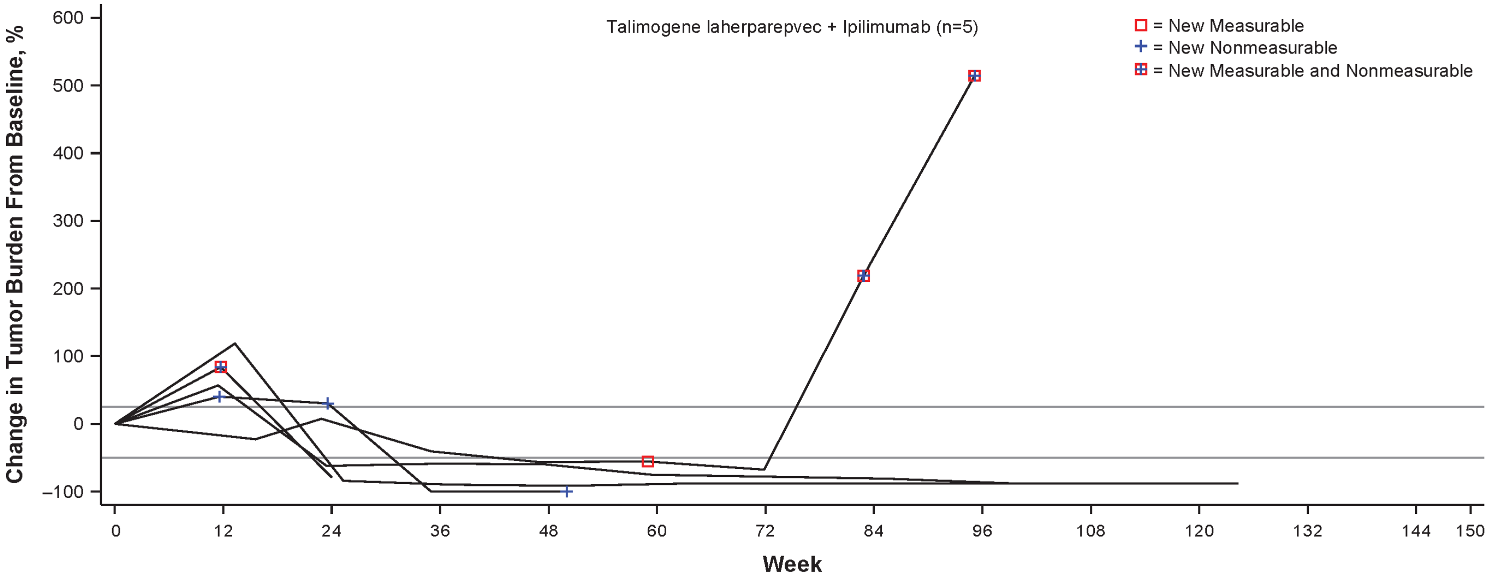

B. Pseudoprogression associated with an increase in existing lesions

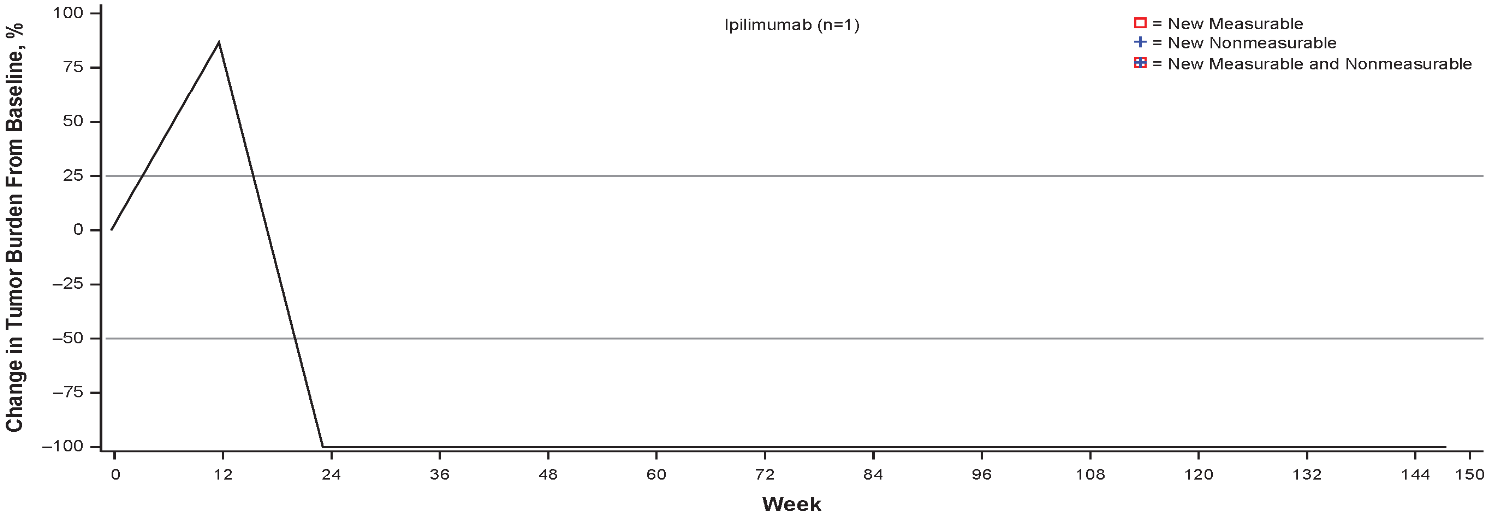

C. Pseudoprogression associated with the development of new lesions

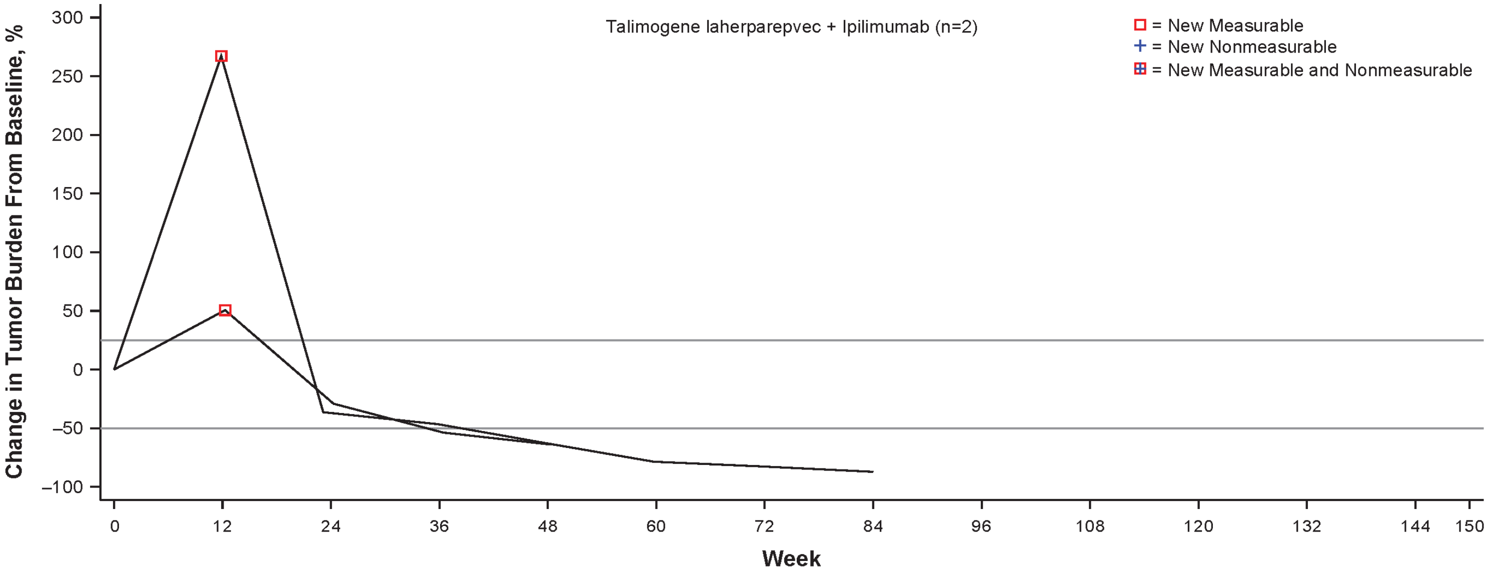

Figure S3. Patterns of response in patients without pseudoprogression. (A) Patients treated with T-VEC plus ipilimumab without pseudoprogression who responded within 6 months following start of treatment; **(B)** patients treated with ipilimumab only without pseudoprogression who responded within 6 months following start of treatment; **(C)** patients treated with T-VEC plus ipilimumab without pseudoprogression who responded after 6 months following start of treatment; **(D)** patients treated with ipilimumab only without pseudoprogression who responded after 6 months following start of treatment.

Figure S3.

A. Patients without pseudoprogression who responded within 6 months following start of treatment

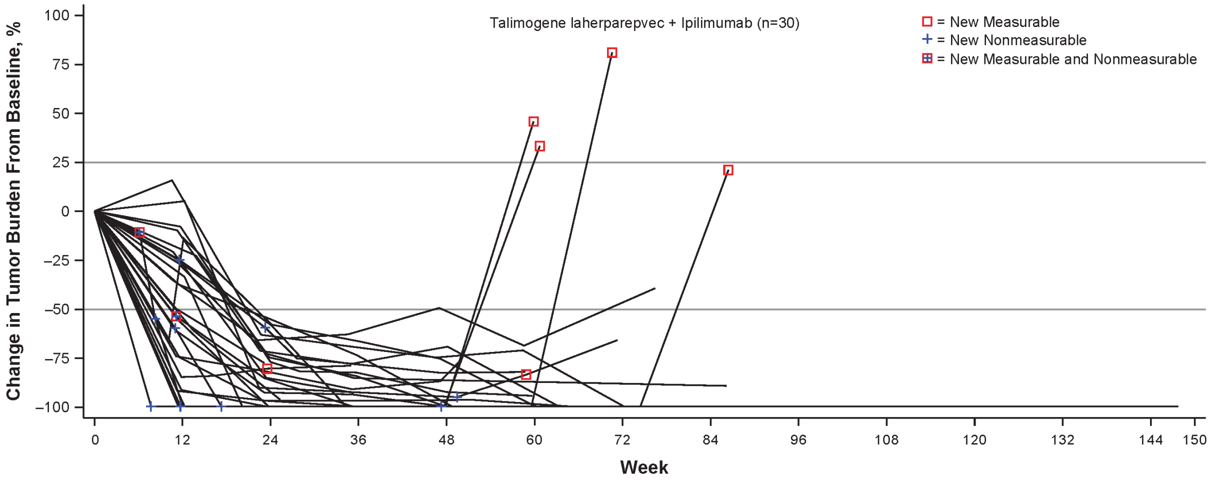

B. Patients without pseudoprogression who responded within 6 months following start of treatment

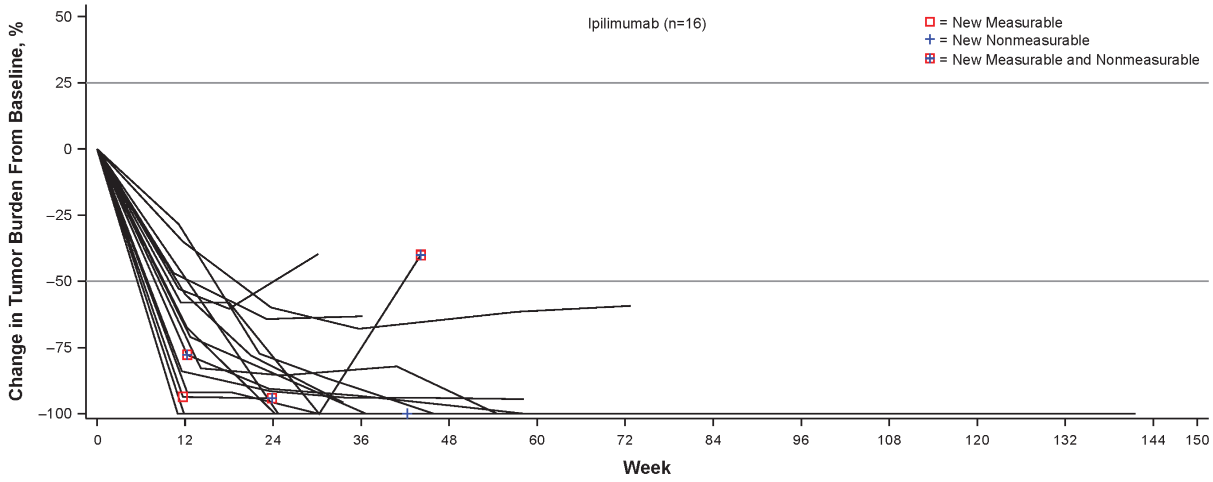

C. Patients without pseudoprogression who responded after 6 months following start of treatment

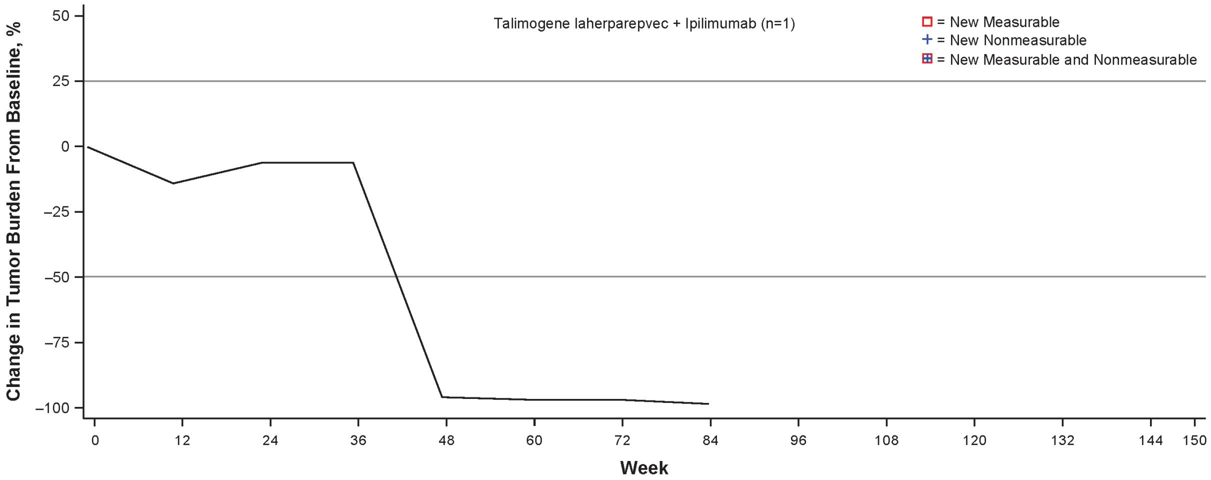

D. Patients without pseudoprogression who responded after 6 months following start of treatment

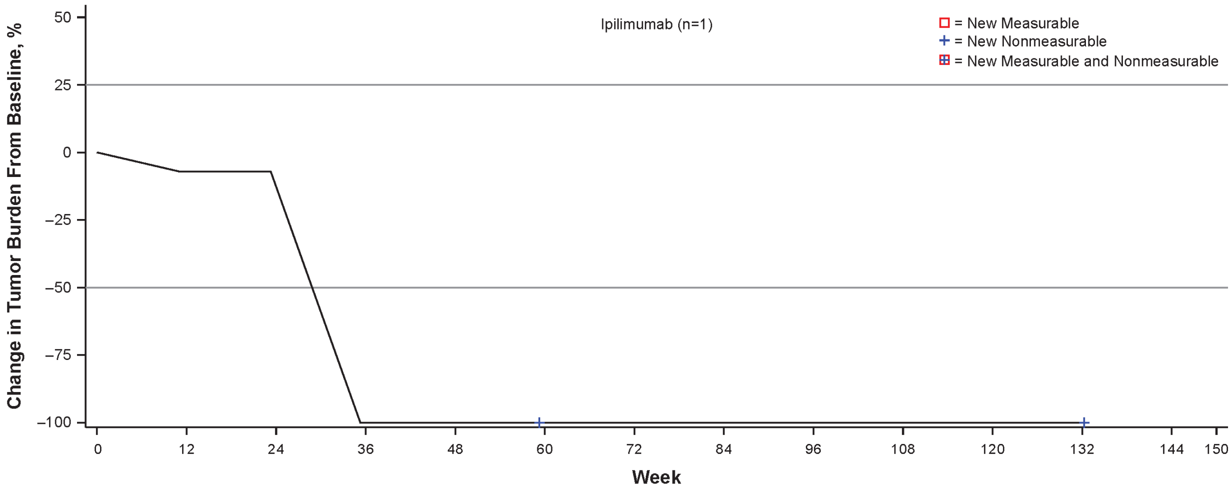

Figure S4. Photographs of patients treated with talimogene laherparepvec plus ipilimumab.

Patient A: A healthy 75-year-old man with a history of cutaneous melanoma of the right shoulder was diagnosed with recurrent, subdermal metastasis of the right deltoid, *BRAF* wild-type. Positron emission tomography (PET) scan showed multiple areas of fludeoxyglucose (FDG) uptake in the spleen consistent with metastatic disease and multiple pulmonary nodules. His baseline deltoid lesion is shown (**1**). Three months into the protocol, the size of the deltoid tumor was significantly decreased, and its vascular supply was diminished (**2**). Six and 10 months into the protocol, there was further decrease in the size of the tumor (**3, 4**). Seventeen months into the protocol, the patient was treated with the last dose of talimogene laherparepvec. Eighteen months into the protocol, the palpable tumor was gone; only white discoloration of the skin remained (**5**). PET scan showed no FDG uptake in the deltoid tumor, stable pulmonary nodules, and improved splenic metastasis. One year later, the patient remains in remission.

Patient B: A 67-year-old patient with stage IIIC melanoma of the left thigh and calf. The patient had had a wide local excision and had been treated with adjuvant radiation therapy that was completed in January 2013. Recurrence of melanoma was noted in May 2013. The patient was subsequently enrolled in the trial. The baseline lesion at enrollment is shown (**1**) before treatment with talimogene laherparepvec plus ipilimumab. The patient achieved a partial response after 24 weeks into the protocol, and the lesion is shown after 58 weeks into the protocol (August 2014; **2**). Further improvement was seen after 130 weeks into the protocol (December 2015; **3**).

Figure S4.

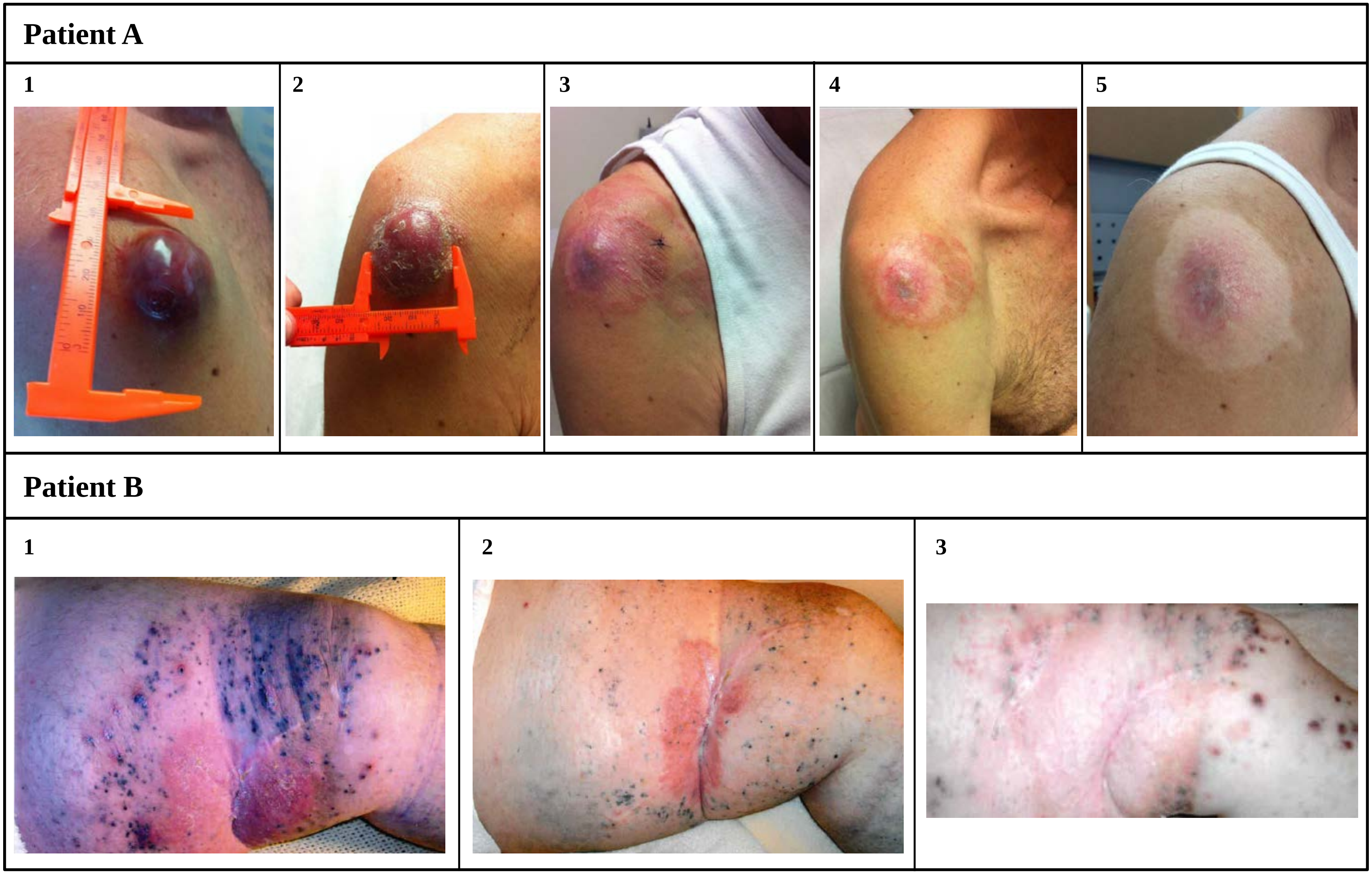

Supplement: Supplementary file 1 — Supplemental Material [file 41416_2019_530_MOESM1_ESM.pdf]
